# Editorial: Role of the Aryl Hydrocarbon Receptor in Immune Modulation

**DOI:** 10.3389/ftox.2022.941665

**Published:** 2022-06-21

**Authors:** Barbara L. F. Kaplan, Carolyn J. Baglole, Courtney E. W. Sulentic

**Affiliations:** ^1^ Center for Environmental Health Sciences, Department of Comparative Biomedical Sciences, College of Veterinary Medicine, Mississippi State University, Mississippi State, MS, United States; ^2^ Department of Medicine, McGill University, Montreal, QC, Canada; ^3^ Department of Pharmacology and Toxicology, Boonshoft School of Medicine, Wright State University, Dayton, OH, United States

**Keywords:** aryl hydrocarbon receptor, immune system, toxicology, mechanism of action, signaling/signaling pathways, immunotoxicology, transcriptional regulation, transcription factors

The aryl hydrocarbon receptor (AhR), originally discovered as a xenobiotic receptor involved in upregulating metabolic enzymes, has become increasingly linked to physiological processes (McMillan and Bradfield, 2007; Okey, 2007; Abel and Haarmann-Stemmann, 2010; Wang et al., 2017; Roman et al., 2018; Rothhammer and Quintana, 2019). The discovery of endogenous, dietary and bacterial-derived ligands for the AhR further supports its involvement in the homeostatic regulation of biological processes (Adachi et al., 2001; Denison et al., 2011; Bessede et al., 2014; Faber et al., 2018; Rannug and Rannug, 2018; Doan et al., 2020). Yet, dysregulation of many of these processes can lead to disease states involving the immune system. Indeed, a toxicological and/or physiological role for the AhR in almost every facet of the immune system has emerged over the past 2 decades. Now, in addition to the AhR being an environmental sensor and mediator of toxicity, this enigmatic protein has emerged as a potential therapeutic target (Murray et al., 2010; Merches et al., 2017; Marafini et al., 2019; Safe et al., 2020; Esser, 2021; Lebwohl et al., 2021). However, many questions remain regarding the interplay between the toxicological and physiological functions of the AhR as well as the molecular mechanisms by which the AhR mediates its pleiotropic effects on immunity. In this special issue, three original research papers explored the role of the AhR in cell- and organ-specific immune responses. Further, two review papers described the role of the AhR as an environmental sensor in the eye as well as the skin and gut.

Over the past decade, the AhR has emerged as a physiological regulator of specific T-cell and innate lymphoid cell (ILC) subtypes (Kiss et al., 2011; Lee et al., 2011; Qiu et al., 2012; Gutiérrez-Vázquez and Quintana, 2018). Until recently, there was negligible information on the AhR in different human B-cell subpopulations. Blevins et al., using human peripheral blood B cells, examined the role of the AhR in a specific B cell subtype, called CD5^+^ “innate-like” B cells (ILB). Although human CD5^+^ B cells are still poorly understood and appear to differ markedly from mouse CD5^+^ B cells, the authors discovered a significant correlation between the percent of CD5^+^ B cells and inhibition of IgM secretion by the high affinity AhR ligand TCDD. Furthermore CD5^−^ conventional B cells were resistant to the inhibitory effect of TCDD. When considering that CD5^+^ B cells appear to play an important protective role against bacterial pathogens, especially in children and the elderly, these results support a potential impairment in this protection upon exposure to environmental AhR ligands.

While the previous publication highlighted a novel role for the AhR in a B cell subtype that bridges adaptive and innate immunity, the publication by Ishihara et al. reinforces the importance of the AhR in innate immunity, and in particular macrophage production of IL-22. The function of IL-22 may be pro- or anti-inflammatory, the nature of which may be context, organ and/or disease-specific. In this study, Ishihara et al. utilized bone marrow-derived macrophages (BMDMs) from wildtype or various knockout mouse models to show that the synergistic induction of IL-22 by AhR ligands and a Toll-like receptor (TLR) ligand was dependent on the AhR and partially dependent on the NF-κB protein RelB. Notably, the induction of IL-22 by TCDD in BMDMs was independent of RORγt and STAT3, which are both required for IL-22 expression in T cells. These results point to striking differences in cellular mechanisms involved in IL-22 production between different immune cell subsets. Another intriguing aspect of this study was the application of particulate matter (PM) collected during wildfires, which increased AhR and NF-κB activity and induced IL-22 in an AhR-dependent manner. Given the importance of the AhR as an environmental sensor, these results could have important implications for susceptibility to develop chronic diseases associated with air pollution exposure.

Like air pollution, cigarette smoke exposure also activates the AhR. In the publication by Guerrina et al., who assessed the chronic effects of cigarette smoke exposure using AhR knockout mice at an age approximate to that of a young adult human, there were more inflammatory cells in the lungs of AhR knockout animals after smoke exposure regardless of the exposure time. At 8-weeks post smoke exposure, this increased inflammatory response caused by AhR deficiency was driven by increased macrophages and multinucleated giant cells (MNGCs), while the enhanced response at 4 months was due primarily to MNGCs. MNGCs may be a potential novel target of the AhR with potential application in suppressing lung inflammation. The origins of MNGCs are not well understood, but they have been identified in other lung diseases. Understanding the effect of AhR in acute versus chronic inflammation, regardless of the inflammogen, will provide guidance on whether targeting AhR therapeutically will be of benefit in lung inflammation.

One difficulty in the development of AhR ligands as therapy is the dichotomy in which many AhR ligands produce either an inflammatory or immunosuppressive response, a topic reviewed by Rannug. In this minireview, which focused on the endogenous ligand FICZ, Rannug suggests that a FICZ/AhR/CYP1A1 feedback loop produces immune homeostasis in a diurnal manner. AhR ligands like FICZ, which is produced in the skin by light exposure, are important to maintaining the dermal barrier as well as IL-22 production from ILC3 cells in the gut, which influences seeding of microbiota on the intestinal epithelium. FICZ is also commonly produced by the microbiota in the skin and gut. The author suggests that the microbiota and exposure to sunlight will lead to fluctuations in AhR ligands. Increased levels of AhR ligands lead to immunosuppression, but these same ligands will increase CYP1A1 via the AhR, which in turn may metabolize these ligands to a lower concentration that promotes immune activation. The net effect of this feedback loop would be oscillations between high and low concentrations of AhR ligands that modulate immune function and maintain immune homeostasis.

Although the AhR has variable expression in various immune cell populations, it is highly and constitutively expressed in barrier organs such as the gut, lung and skin. Another organ also exposed to the environment, but understudied in relation to AhR function, is the eye. Thus, Hammond et al., provides a timely and important overview of studies supporting a role for AhR in several ocular diseases. Physiologically, the retina of the eye is one of the most oxygen-consuming tissues making it susceptible to oxidative stress, and the ocular surface is at risk to toxicant insults from smoke and/or other sources of particulate matter (e.g., air pollution). The authors discuss evidence in animal and human studies that AhR activation is one possible therapeutic strategy to reduce inflammation in a wide variety of ocular diseases.

Together these papers suggest that the potential for the AhR to be a therapeutic target for immune-mediated diseases likely depends on several factors, including the disease state and/or the cell types involved in disease induction. As shown in this issue, some B-cell subtypes might be refractory to modulation via AhR (Blevins et al.). On the other hand, MNGCs are novel targets of AhR (Guerrina et al.). The studies also highlight the important role that AhR plays in IL-22 induction and immune homeostasis, which depending on its context, can be immune enhancing or suppressing (Ishihara et al.; Rannug). Despite all we have discovered about the AhR and its role in immune modulation, more studies are needed to decipher how best to target it for therapeutic benefit.
